# Ascorbic Acid (Vitamin C) as a Cosmeceutical to Increase Dermal Collagen for Skin Antiaging Purposes: Emerging Combination Therapies

**DOI:** 10.3390/antiox11091663

**Published:** 2022-08-26

**Authors:** Yong Chool Boo

**Affiliations:** 1 Department of Molecular Medicine, School of Medicine, Kyungpook National University, 680 Gukchaebosang-ro, Jung-gu, Daegu 41944, Korea; ycboo@knu.ac.kr; Tel.: +82-5-3420-4946; 2 BK21 Plus KNU Biomedical Convergence Program, Department of Biomedical Science, The Graduate School, Kyungpook National University, 680 Gukchaebosang-ro, Jung-gu, Daegu 41944, Korea; 3 Cell and Matrix Research Institute, Kyungpook National University, 680 Gukchaebosang-ro, Jung-gu, Daegu 41944, Korea

**Keywords:** ascorbic acid, vitamin C, collagen, skin aging, antiaging, amino acid, glycinamide, glycine, cosmetic, cosmeceutical

## Abstract

Ascorbic acid (AA) is an essential nutrient and has great potential as a cosmeceutical that protects the health and beauty of the skin. AA is expected to attenuate photoaging and the natural aging of the skin by reducing oxidative stress caused by external and internal factors and by promoting collagen gene expression and maturation. In this review, the biochemical basis of AA associated with collagen metabolism and clinical evidence of AA in increasing dermal collagen and inhibiting skin aging were discussed. In addition, we reviewed emerging strategies that have been developed to overcome the shortcomings of AA as a cosmeceutical and achieve maximum efficacy. Because extracellular matrix proteins, such as collagen, have unique amino acid compositions, their production in cells is influenced by the availability of specific amino acids. For example, glycine residues occupy 1/3 of amino acid residues in collagen protein, and the supply of glycine can be a limiting factor for collagen synthesis. Experiments showed that glycinamide was the most effective among the various amino acids and amidated amino acids in stimulating collagen production in human dermal fibroblasts. Thus, it is possible to synergistically improve collagen synthesis by combining AA analogs and amino acid analogs that act at different stages of the collagen production process. This combination therapy would be useful for skin antiaging that requires enhanced collagen production.

## 1. Introduction

L-Ascorbic acid (AA) (vitamin C), discovered during research into the causes and treatment of scurvy, plays an essential role in human physiology [1]. The biochemical function of AA is mostly related to its redox activity [2,3]. In other words, AA has the characteristic of being easily oxidized by releasing electrons in an aqueous solution, so it can act as a water-soluble antioxidant that removes reactive oxygen species (ROS) or free radicals [4]. It also acts as a cofactor that assists catalytic activity by reducing metal ions in the active site of the enzyme [5,6]. AA also performs other essential functions for cell physiology and human health, such as preserving important biomolecules in a reduced state [7].

Collagen is the most abundant protein in our body and is a major component of the extracellular matrix (ECM) [8,9]. Collagen molecules exist in a triple helix structure in which three primary protein strands are twisted, and the amino acid sequence of the collagen protein is typically glycine-proline-X or glycine-X-hydroxyproline, where X may be any amino acid other than glycine, proline, or hydroxyproline [10,11]. Twenty-eight types of collagen proteins are known so far, and there are some differences in amino acid composition and sequence [12,13]. Transforming growth factor (TGF)-β1 plays an important and diverse role in the synthesis of collagen [14], and the degradation of collagen is mediated by matrix metalloproteinases (MMPs) [15,16].

The skin is an organ that is exposed to various external environmental factors [17]. In the process of interaction of internal and external factors, the skin undergoes various types of functional and structural degeneration associated with aging [18]. Skin collagen is composed of 80–90% type I, 8–12% type III, and 5% type V [19]. Insufficient collagen synthesis and maturation due to genetic factors or nutritional deficiencies, and excessive increase in collagen synthesis due to an autoimmune response, can cause skin diseases [20]. Extrinsic and intrinsic skin aging commonly leads to changes in the amount, type, and structure of collagen [21,22], which is accompanied by a decrease in TGF-β1 and an increase in MMPs [23,24].

AA is known to affect multiple stages in collagen production. As an essential cofactor of prolyl hydroxylase and lysyl hydroxylase, AA plays a critical role in the maturation of collagen at the post-translational stage [25]. AA has been observed to stimulate procollagen I and III gene transcription in cells [26,27]. The level of AA in the skin cells is closely associated with the quantitative and structural integrity of dermal collagen, and AA deficiency causes skin aging [28,29]. Thus, the external supplementation of AA is one of the attractive strategies for skin antiaging [29]. On the other hand, AA has limitations in that it is easily oxidized and difficult to absorb into the skin [30].

The purpose of this review is to examine the potential and applications of AA as a cosmeceutical active ingredient to promote collagen production for skin antiaging purposes. First, we will briefly discuss the biochemical basis of AA associated with collagen metabolism. Then, we will review the evidence for dermal collagen-enhancing and skin antiaging effects of AA. Finally, we will introduce emerging approaches to overcome the shortcomings of AA and maximize its clinical efficacy. It is hoped that this review will help to derive future tasks necessary for the development of advanced skin antiaging strategies.

## 2. Biochemical Properties of AA

AA is synthesized through multiple pathways in plants and some animals [31,32]. However, humans cannot make AA because they lack functional gulonolactone oxidase catalyzing the last step of AA synthesis [33]. So AA is a kind of vitamin to humans that must be ingested from the outside. Externally sourced AA and its oxidized form, dehydroascorbate (DHA), can enter cells through specific mechanisms of transport [34]. The sodium-dependent vitamin C transporter (SVCT) 1 and SVCT 2 of the human solute carrier (SLC) 23 family mediate the active transport of AA [35]. The facilitated transport of DHA is mediated by GLUT 1, GLUT 3, and GLUT 4 of the glucose transport (GLUT) family [36]. The hydrolysis of DHA yields 2,3-diketo-L-gulonate, which is further hydrolyzed to oxalate and L-erythrulose, decarboxylated to form L-xylonic acid or L-lyxonic acid, or degraded to other small molecules [32].

AA is a type of diacid and is present in the form of ascorbate mono-anion (Asc^−^) in a neutral aqueous solution [37]. Oxidation reactions of AA produce monodehydroascorbate (ascorbyl radical, Asc^•^) and DHA. AA is easily oxidized by reaction with oxygen at high temperatures [38] and the reaction is facilitated by transition metal ions [39]. AA rapidly reacts with various types of ROS and free radicals to scavenge them, and it is oxidized to ascorbyl radical [3]. The reaction of AA with singlet oxygen produces DHA and hydrogen peroxide [40]. By scavenging the lipid radicals mediating the chain reaction of lipid peroxidation in the cell membrane, α-tocopherol acts as a chain-breaking antioxidant [41]. The generated α-tocopheroxyl radical reacts with AA to regenerate α-tocopherol, and, as a result, AA is oxidized to ascorbyl radical.

AA functions as an essential cofactor of certain metalloenzymes, such as 2-oxoglutarate-dependent dioxygenases [5]. 2-Oxoglutarate-dependent dioxygenases have iron ions at their active sites, and the reaction requires molecular oxygen and 2-oxoglutarate (also called α-ketoglutarate) as co-substrates [42]. The enzymes play an important role in collagen metabolism and many other biochemical processes, such as development, transcriptional regulation, and the modifications of nucleic acid, hormones, fatty acids, and antibiotics [43]. In the catalytic process of the enzymes, by returning the oxidized iron ion to the reduced state, AA affects the activity of the enzyme and various physiological processes. Of course, in the course of this reaction, AA is oxidized.

AA can be regenerated from ascorbyl radical and DHA chemically and enzymatically. Two molecules of ascorbyl radical are disproportionated to form DHA and AA. Ascorbyl radical can be directly reduced to AA by the enzymes with monodehydroascorbate reductase activity using half equivalent of reduced nicotinamide adenine dinucleotide (NADH) + H^+^ [44]. DHA can be reduced to AA by the enzymes with dehydroascorbate reductase activity in the concert of the oxidation of glutathione (GSH) to glutathione disulfide (GSSG) [45].

## 3. AA in Collagen Metabolism

Collagen is synthesized in cells and secreted to the outside through several complex steps [46]. In an early step, collagen genes are transcribed into mRNA in the nucleus, which is transported to the cytoplasm and translated by ribosomes to synthesize a pre-procollagen molecule [47]. The pre-procollagen is transported to the endoplasmic reticulum where the *N*-terminal signal peptide is removed, proline and lysine residues are hydroxylated, and specific hydroxyl groups of lysine residues are glycosylated by galactose and glucose [48]. Three procollagen molecules are left-twisted into a triple helix by zipper-like folds and transported to the Golgi apparatus [49]. After additional modifications in the Golgi apparatus, the triple helix of procollagen molecules is assembled into secretory vesicles and transported out of the cell [50]. Both ends of the triple helix are removed to generate a tropocollagen molecule, and covalent bonds are formed between the tropocollagen molecules, producing collagen fibrils which are further bundled into collagen fibers [51].

As one of the posttranslational modifications of the protein, several amino acid residues such as proline, lysine, asparagine, aspartate, and histidine are hydroxylated [52]. In the case of collagen, the content of proline and lysine is particularly high, and the hydroxylation of these amino acid residues is important for stabilizing the structure of collagen proteins. The hydroxylation of proline residues occurs mainly at the γ-C atom and sometimes at the β-C atom, and hydroxylation of the lysine residue occurs at the δ-C atom [53,54]. These reactions are catalyzed by enzymes called prolyl 4-hydroxylase, prolyl 3-hydroxylase, and lysyl 5-hydroxylase, respectively [55]. These 2-oxoglutarate-dependent dioxygenases require AA for proper function, and thus AA deficiency can cause defects in collagen maturation [56].

In early studies, it was observed that AA enhances the production of collagen at pharmacological doses, but its mechanism of action and physiological significance are controversial. AA was shown to selectively increase collagen protein synthesis without increasing noncollagenous protein synthesis in cultured human dermal fibroblasts [57,58]. The effect was associated with elevated mRNA levels of type I and type III procollagen [26,59]. Pinnel et al. [59] proposed a hypothesis that procollagen accumulated in the cell inhibits the translation of procollagen synthesis, and that AA releases the translational inhibition by promoting the hydroxylation and secretion of procollagen, which results in increased collagen gene transcription.

Other research groups have proposed that lipid peroxidation mediates the increase in collagen gene expression stimulated by AA [60,61]. The addition of malondialdehyde, a product of lipid peroxidation, increased collagen gene expression, and α-tocopherol, a chain-breaking antioxidant, inhibited collagen gene expression [60,61]. However, cell-impermeable iron chelators, such as desferrioxamine and ethylenediaminetetraacetic acid, did not reduce AA-induced collagen synthesis although they abolished AA-mediated lipid peroxidation [62]. Thus, it is uncertain whether the antioxidant or prooxidant properties of AA are associated with its stimulatory action in collagen gene expression.

It would be interesting to examine whether the AA-induced collagen gene expression is associated with the modulation of 2-oxoglutarate-dependent dioxygenases. If so, many target proteins of 2-oxoglutarate-dependent dioxygenases, such as hypoxia-inducible factors (HIFs), could be considered potential regulators of collagen gene expression [52,63]. Separately, it is worth examining whether AA activates the specific protein 1 (Sp1) family, as these transcription factors are known as positive regulators of collagen gene expression [64,65]. Identification of the molecular mechanism underlying AA-induced collagen gene expression will contribute to the development of new strategies to enhance collagen production.

## 4. Clinical Evidence for Dermal Collagen-Enhancing and Skin Antiaging Effects of AA

Do photoaging and the natural aging of the skin accompany changes in AA levels in the skin? Rhie et al. [66] reported the changes in the levels of antioxidant enzymes and antioxidants in the epidermis and dermis of sun-exposed forearms and sun-protected upper-inner arms in young (average 21.0 years old) and old (average 76.1 years old) Koreans. The epidermal and dermal AA levels of the photoaged skin were 69% and 63% of the levels of young skin, respectively, and the epidermal and dermal AA levels of the naturally aged skin were 61% and 70% of the levels of young skin, respectively. Thus, it is suggested that both photoaging and natural aging can lower AA levels in the epidermis and dermis, and external supplementation might help retard skin aging.

Clinical trials have been conducted on whether the topical application of AA can increase skin collagen. In a single-blind, randomized, split-face, placebo-controlled clinical study, 10 postmenopausal women applied on the dorsal side of the upper forearm, a stabilized W/O emulsion cream containing 5% AA on one side and placebo on the other side, at night, once daily for 6 months [67]. The mRNA levels of collagen type I and type III in the skin biopsy were increased in AA-treated groups by 25% and 21%, respectively. However, in this study, no significant difference was observed between the AA-treated group and the control group in the concentration of collagen extractable from the skin.

In another clinical trial, 5% AA cream and one of several cosmeceuticals were applied twice a day to the extensor surface of one forearm for 2 weeks, and the level of procollagen I in the skin biopsy was analyzed [68]. Statistical analysis of all 19 subjects who applied 5% AA cream did not show a significant increase in procollagen level compared to the baseline before treatment. However, when the subjects were divided into a group with a low baseline procollagen I level (*n* = 9) and a group with a high baseline procollagen I level (*n* = 10), the former group showed a significant increase in procollagen I level with AA cream application. These studies suggest that AA helps to increase collagen in the skin, especially or only when there is a lack of AA or collagen.

Clinical evidence has been reported that topical treatment of AA alleviates the symptoms of skin aging. In a randomized, double-blind, placebo-controlled study enrolling 19 healthy female volunteers, topical application of 5% AA cream on the sun-exposed upper chest and forearm once a day for 6 months significantly increased the density of skin microrelief, decreased the deep furrows, and improved ultrastructure of the skin, compared to the excipient treatment [68]. In a split-face study enrolling 20 women subjects with photo-aged skin, topical treatment of 23.8% AA serum with iontophoresis on one side of the face once a day for 2 weeks improved hyperpigmentation, surface roughness, and fine lines on the treated side significantly compared to the other side spared for participants’ self-control [69].

The antiwrinkle effect of an AA-loaded dissolving microneedle patch was evaluated in a double-blind, split-face, placebo-controlled clinical study by applying it around crow’s feet on one side of the face and a placebo patch on the other side, for 12 weeks in 23 subjects with notable wrinkles near the eyes [70]. The AA-loaded dissolving microneedle patch significantly reduced wrinkles in the crow’s feet area without causing skin irritation and sensitization problems.

In a double-blind, prospective, randomized clinical trial, patients were subjected to the laser skin resurfacing procedure followed by topical treatment of 200 mg AA with or without a cosmeceutical containing growth factors [71]. Three months after the treatments, there was a significant reduction in skin roughness and the average depth of periorbital wrinkles in both groups, and the group (*n* = 75) treated with AA plus growth factors showed better results compared with the group (*n* = 74) treated with AA alone.

The results of these studies suggest that cosmetic application of AA can help alleviate the symptoms of skin aging, but the number of studies is not considered sufficient to confirm it.

## 5. Clinical Studies Using a Combined Composition of AA and Other Active Ingredients

In cosmetics, many attempts are made to enhance efficacy by combining several active ingredients. We would like to introduce clinical trials that have evaluated the collagen enhancement and skin anti-aging effects of a combined composition of AA and other active ingredients.

In a double-blind placebo-controlled randomized study involving 159 volunteers, increased deposition of collagen fibers in the dermis and reduction in the depth of facial wrinkles were demonstrated in post-menopausal women who consumed a drink containing soy isoflavones, lycopene, AA, and α-tocopherol with a capsule containing fish oil, daily for 14 weeks [72].

A solution containing AA (5%), proteoglycans (3%), Rosa moschata oil (0.3%), polysorbate 80 (1%), propylene glycol (70%), water, perfume, and preservatives (sodium methylparaben, sodium ethylparaben, and sodium propylparaben; 0.14%), was applied on the face every evening for 60 days in a clinical study involving 60 Caucasian female healthy individuals [73]. The ultrasonographic image analysis showed that the treatment significantly increased the high echogenic pixels that are associated with collagen levels.

In an ex vivo experiment using abdominal skin samples obtained from healthy women, topical application (2 mg cm^−2^) of a serum containing AA, ergothioneine, a proteoglycan-stimulating peptide (tetradecyl aminobutyroyl valylaminobutyric urea trifluoroacetate), soluble proteoglycans, and low molecular weight hyaluronic acid for 10 days significantly prevented the decreases in the levels of collagen, elastin, and proteoglycan induced by daily irradiation with UV/visible/infrared rays (10 J cm^−2^) or by daily treatment with hydrocortisone (10 µg mL^−1^) [74].

A split-face, randomized controlled trial involving 50 female volunteers demonstrated the skin antiaging effects (improvement in skin color, elasticity, radiance, smoothness, scales, and wrinkles) of an encapsulated serum that contains AA (20%), α-tocopherol, and *Rubus idaeus* leaf cell culture extract, which was topically applied on one side of the face for 2 months [75].

In an open-label study, application of a serum comprising AA (15%), α-tocopheryl acetate, palmitoyl tripeptide-38 (5 ppm), and other ingredients to the face once daily for 56 days decreased skin roughness by 8% and improved skin tone (redness, 9% decrease; homogeneity, 8% increase) [76].

A formulation containing AA (10%), biopeptides (rice and lupin), hyaluronic acid, and Vichy volcanic mineralizing water with no preservatives, was tested in three consecutive clinical studies and shown to enhance skin cell turnover compared to untreated skin (cumulative fluorescence score of dansyl chloride probe, 59.6 versus 64.9) (for 3 weeks in 32 subjects); reduce the clinical grades of wrinkles at crow’s feet, forehead, and nasolabial fold by 9%, 11%, and 5.2%, respectively (for 4 weeks in 40 subjects); and decrease the number of crow’s-foot wrinkles by 11.5% and their maximal vertical distance between the highest peak and lowest valley by 13% (after 29 days compared to the baseline in 52 subjects) [77].

These clinical results suggest that several combination compositions containing AA may help increase collagen in the skin and delay skin aging. Since the tested compositions contained multiple active ingredients including AA, there is a limitation in that it cannot be confirmed whether the efficacy is due to AA or other ingredients.

## 6. Various Approaches to Maximize the Efficacy of AA

AA is chemically active and unstable in aqueous media. It is easily oxidized or decomposed in an oxygen-dependent or -independent manner, and the reaction rate increases with oxygen concentration, temperature, and metal ion concentration [3,39]. The decomposition rate of AA is pH-dependent in an aqueous solution and AA is decomposed faster at pH 5.6 than at pH 1.0–4.4 or pH 6.8–8.4 [78]. The half-lives of AA in water-in-oil emulsions were estimated to be 20 days and 10 days at 25 °C and 45 °C, respectively [79]. AA is very prone to photooxidation [80].

AA is a hydrophilic compound and has limited skin absorption. In an experiment using excised hairless mouse skin, the permeation rate of AA through the skin with the stratum corneum removed increased about 10 times compared to that of the intact skin, so the main barrier to skin absorption of AA is the stratum corneum [81].

Because cosmetics are continuously used for a relatively long period under aerobic and moisturized conditions, the chemical stability of active ingredients is important in addition to their efficacy and safety. Furthermore, since cosmetics are applied topically to the skin, effective absorption of active ingredients through the skin is also important. From this point of view, AA is a material with shortcomings as a cosmeceutical [30].

**Figure 1:**
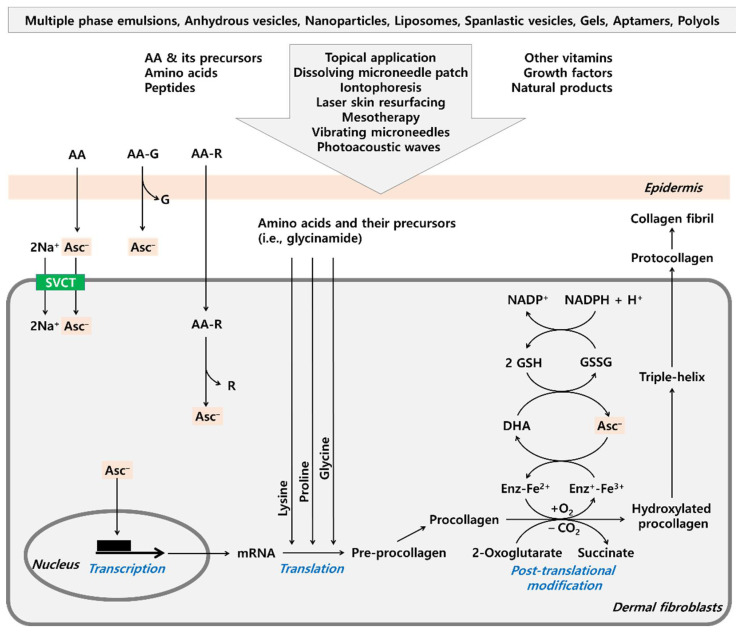
Various strategies to enhance the efficacy of ascorbic acid (AA) increasing dermal collagen. AA is present as ascorbate mono-anion (Asc^−^) at neutral pH. The efficacy of AA may be synergistically enhanced by combined formulation with other active ingredients, such as other vitamins, amino acids, peptides, growth factors, and natural products. The incorporation of AA into multiple phase emulsions, anhydrous vesicles, nanoparticles, liposomes, spanlastic vesicles, gels enhances the stability and skin permeation of AA. Aptamers and polyols help stabilize AA. To enhance skin permeation of AA, its topical application can be combined with other procedures, such as iontophoresis, laser skin resurfacing, mesotherapy, and the use of vibrating microneedles and photoacoustic waves. AA-loaded dissolving microneedle patches are also useful for this purpose. AA glycosides and hydrophobic AA precursors can be used instead of AA for higher stability in cosmetic products and a more effective supply of AA to skin cells. AA glycosides (AA-G) are enzymatically hydrolyzed to release AA as they pass through the skin. While AA enters cells through the SVCT family, hydrophobic AA precursors (AA-R) can enter cells through simple diffusion and be converted to AA by intracellular enzymes. In the process of collagen production, AA stimulates the mRNA expression of procollagen genes at the transcription stage. AA also enhances the hydroxylation of procollagen at the post-translational modification stage, by acting as a cofactor of 2-oxoglutarate-dependent dioxygenase (Enz). AA reduces the iron ions at the active sites of the enzyme while it is oxidized to dehydroascorbate (DHA). DHA is enzymatically reduced to AA in a reaction coupled with the oxidation of glutathione (GSH) to glutathione disulfide (GSSG). GSSG is reduced to GSH in a reaction coupled with the oxidation of nicotinamide adenine dinucleotide phosphate hydrogen (NADPH) to nicotinamide adenine dinucleotide phosphate (NADP^+^). Amino acids can serve as the building blocks of proteins needed at the translation stage. In particular, glycine, proline, and lysine, or their precursors, can help facilitate collagen production.

Various approaches are being developed to overcome the shortcomings of AA as a cosmeceutical and to maximize its clinical efficacy, and some of them are illustrated in Figure 1.

### 6.1. AA Derivatives with Added Advantages

**Figure 2:**
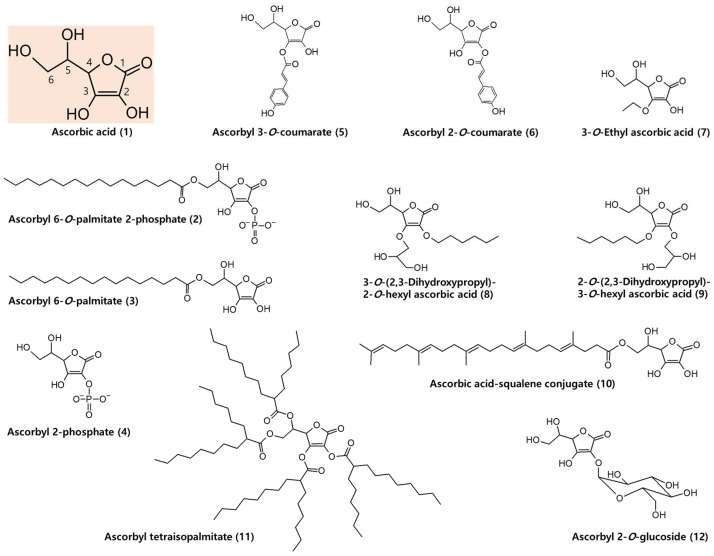
Chemical structures of ascorbic acid and its derivatives.

Various AA derivatives with excellent safety, biological activity, and stability have been developed [82]. In Figure 2, the chemical structure of AA (1) and some of its derivatives are shown.

Hydrophobic AA precursors can pass through the cell membrane and enter the cell by simple diffusion and can be enzymatically hydrolyzed in the cell to regenerate AA. For example, externally added ascorbyl 6-*O*-palmitate 2-phosphate (trisodium salt) (2) increased intracellular AA concentration more effectively than external AA (4.1 fold versus 2.3 fold) in human dermal fibroblasts (TIG118) [83]. The increase of intracellular AA by external AA was inhibited by phorbol 12-myristate 13-acetate and glucose, which are inhibitors of SVCTs and GLUTs, respectively, but the increase by ascorbyl 6-*O*-palmitate 2-phosphate was not affected, suggesting it is absorbed through simple diffusion. Ascorbyl 6-*O*-palmitate 2-phosphate was converted to AA by intracellular enzymes mainly through ascorbyl 6-*O*-palmitate (3) and partially through ascorbyl 2-phosphate (4).

In our study [84], ascorbyl 3-*O*-coumarate (5) and ascorbyl 2-*O*-coumarate (6) were synthesized as multifunctional cosmeceutical agents to inhibit melanin synthesis and increase collagen synthesis. At 100 μM, ascorbyl 3-*O*-coumarate and ascorbyl 2-*O*-coumarate decreased the melanin content of human dermal melanocytes by 65% and 59%, respectively. At 100~300 μM, the compounds augmented collagen synthesis in human dermal fibroblasts by 120~144% and 125~191%, respectively. They increased procollagen type I C-peptide release, and ascorbyl 2-*O*-coumarate decreased MMP1 level, indicating that they might regulate collagen metabolism by multiple mechanisms.

3-*O*-Ethyl ascorbic acid (7) is a stabilized form of AA, and skin permeation can be enhanced by using a proper single solvent, such as glycerol, propylene glycol, and 1,2-hexanediol [85]. A serum containing 3-*O*-ethyl ascorbic acid (30%), lactic acid (1%), and other ingredients has been tested for safety and biological activities in HaCaT keratinocytes, human dermal fibroblasts, reconstructed human epidermis, and reconstructed human pigmented epidermis [86]. The serum reduced ultraviolet radiation (UV)-B-induced DNA damage and melanin synthesis and increased collagen production.

Twenty-eight alkyl glyceryl AA derivatives were synthesized and tested in theophylline-stimulated B16 melanoma 4A5 cells to determine if they inhibit melanogenesis [82]. The longer the alkyl chains, the greater the melanogenesis inhibitory effects, but cytotoxicity also increased. 3-*O*-(2,3-dihydroxy propyl)-2-*O*-hexyl ascorbic acid (8) and 2-*O*-(2,3-dihydroxy propyl)-3-*O*-hexyl ascorbic acid (9) were selected as the optimized compounds for their inhibitory activities and low toxicities. Both compounds were more stable than AA.

AA was conjugated to squalene, a natural lipid of the skin, and the biological activity of AA-squalene conjugate (10) was investigated ex vivo in human skin explants [87]. AA-squalene conjugate (3%) significantly increased epidermal thickness and the expression of collagen III and glycosaminoglycans after application for 10 days, to a higher extent than free AA and ascorbyl 6-*O*-palmitate. It also increased the transcription of various ECM components in the epidermis and dermis than free AA and ascorbyl palmitate as well as the negative control.

Ascorbyl tetraisopalmitate (11), a liquid form AA precursor, protected UV-A-induced cytotoxicity in human keratinocytes, enhanced collagen production, and repressed MMP2 and MMP9 activities in human fibroblasts [88]. Its anti-wrinkle effect was evaluated in double-blind, randomized, placebo-controlled, split-face clinical trials, by applying either 1%, 2%, or 3% ascorbyl tetraisopalmitate cream on the periorbital area on one side of the face, and placebo cream on the other side of the face, for 8 weeks in 3 groups of 23 female subjects [89]. Each ascorbyl tetraisopalmitate cream significantly reduced periorbital wrinkles compared to the placebo although dose-dependency was not observed.

Ascorbyl 2-*O*-glucoside (12) stimulates collagen synthesis and exhibits antioxidant and antisenescence effects in human dermal fibroblasts [90,91]. It is being used in cosmetics for various purposes, such as skin antiaging [92]. Because this compound has strong hydrophilicity, its skin permeability is lower than that of AA [93]. This compound is relatively stable in the cosmetic formulation and, when topically applied, it can be enzymatically hydrolyzed in the skin, producing AA [93,94].

In summary, certain hydrophobic AA precursors could be more effective than AA itself in increasing the concentration of AA in the cytoplasm because it is easy to pass through the cell membrane [83,88]. Topically applied AA glycosides can serve as a reservoir for AA because they continuously release AA by enzymatic hydrolysis in the skin [94]. Multifunctional hybrid compounds developed by covalently combining two substances with different biological activities and physicochemical properties would have added benefits [84,87].

### 6.2. Formulations to Improve the Stability and Skin Absorption of AA

Multiple phase emulsions of AA have been prepared for the enhanced stability and slow controlled release of the drug [95]. The oil/water/oil emulsions formulated from non-ionic siliconized surfactants, sorbitan derivatives, and co-surfactants such as polyglycerol derivatives, using a two-step procedure, significantly increased the stability of AA, possibly through the entrapment of AA inside the reverse micelles surrounded by hydrophilic heads of surfactant. The in vitro release studies using Franz diffusion cell showed that about 14% of AA was released from the multiple phase emulsions in the first 4 h period with the release profile following zero-order kinetics.

Anhydrous formulations containing microfine particles of AA in an anhydrous oil/wax vehicle or an anhydrous silicone/oil/wax vehicle have been prepared to provide it greater stability than an aqueous vehicle [96]. The anhydrous formulations applied topically onto freshly excised human abdominal skin increased the production of collagen types I and III and cytokeratin within 48 h.

Aptamers are single-stranded DNA or RNA-based oligonucleotides that show specific binding affinity to a wide range of molecules [97]. The complex formation of AA with an optimized DNA aptamer enhanced its stability [98]. The complex reduced MMP1 expression and increased collagen synthesis in human dermal fibroblasts in vitro and improved wrinkles of the crow’s feet in a clinical test undertaken with 22 female subjects for 8 weeks.

Some polyols used in cosmetics can extend the half-lives of AA, and the inclusion of glycerin improved the stability of AA in the water-in-oil emulsion more effectively than the inclusion of propylene glycol [79]. The photooxidation of AA was significantly slower in a gel preparation based on polyacrylic acid and glycerol compared to a single-component solution [99].

Various types of nanoparticles have been developed for the efficient and controlled delivery of AA and its derivatives [100,101]. Stevanovic et al. produced copolymer poly (D, L-lactide-co-glycolide) (DLPLG) nanoparticles and encapsulated up to 15% of AA in the polymer matrix through homogenization of water and organic phases while preserving the spherical shape, size, and uniformity of nanoparticles [100]. Duarah et al. loaded AA into ethyl cellulose nanoparticles by the solvent evaporation method and formulated them with hydroxypropyl methyl cellulose gels [101]. The optimized gel exhibited sustained AA release over 8 h in vitro.

Phosphatidylcholine liposomal formulation enhanced the penetration of sodium ascorbate ex vivo across the abdominal skin of human patients [102]. The sodium ascorbate-loaded phosphatidylcholine liposomes diffuse better through the dermis than the epidermis. The liposomes exhibited antioxidant and anti-inflammatory properties in human skin exposed to UV-A/UV-B radiation. The incorporation of AA into a negatively charged liposome increased the stability, skin permeation, and cell absorption of AA, and exhibited a higher collagen synthesis promoting effect than AA solutions [25]. The flux of AA through an excised pig ear skin was affected by liposome composition, and the presence of cholesterol and surface charge contributed to an increase in the amount of AA across the skin. This formulation promoted a 7-fold increase in drug flux compared to the free AA solution.

Starr et al. analyzed the three-dimensional skin permeation profile of AA and ascorbyl 2-*O*-glucoside in aqueous solutions or supramolecular hydrogel formulations in ex vivo porcine tissues using time of flight secondary ion mass spectrometry (ToF-SIMS) [93]. The results showed that the skin absorption of AA and ascorbyl 2-*O*-glucoside was greatly enhanced when they were applied in the form of a supramolecular hydrogel containing an amphiphilic gemini imidazolium-based surfactant. Skin absorption of ascorbyl 2-*O*-glucoside was less than AA, but the former compound liberated the latter in the skin tissue, supporting the utility of ascorbyl 2-*O*-glucoside in continuously supplying AA for an extended time.

For the topical delivery of 3-*O*-ethyl l-ascorbic acid, binary and ternary solvent systems have been developed by Iliopoulos et al. [103]. A binary mixture of propylene glycol and propylene glycol monolaurate effectively enhanced skin permeation of 3-*O*-ethyl ascorbic acid across the porcine skin compared with individual solvents. The optimized ternary solvent system containing propylene glycol: propylene glycol monolaurate: isopropyl myristate promoted up to 70.9% skin delivery of 3-*O*-ethyl ascorbic acid.

AA-loaded spanlastic vesicles prepared using an optimized ratio of span 60 and tween 60 (5:1) showed high entrapment efficiency, enhanced stability, good physicochemical stability, and improved drug penetration into the stratum corneum [104]. Topical application of AA-loaded spanlastic vesicles suppressed MMP2 and MMP9 expression in the skin and normalized epidermal thickness and the densely arranged dermal collagen fibers in rats exposed to UV-B radiation more effectively compared to AA solution.

In summary, the greatest challenge for cosmetic applications of AA is to maintain stability and improve delivery to the target site, which has been partially overcome by various advanced formulation technologies. In fact, these technologies are being applied to cosmetics [105]. In order to expand the industrial application of AA, continuous research and development for optimal formulations are required.

### 6.3. Use of Medical Devices to Enhance Skin Absorption of AA

To enhance skin permeation of AA, topical application can be combined with other procedures, such as iontophoresis and laser skin resurfacing.

Ebihara et al. used radioactive [^14^C]-AA to trace iontophoresis-assisted drug absorption in rat skin [106]. The use of iontophoresis after topical application of AA markedly enhanced percutaneous drug absorption compared to a simple topical application. Radioactive tracer in the dermis reached a maximum level at 1 h after application. Hori et al. demonstrated that iontophoresis increased collagen production in rat skin as determined by hydroxyl proline level [107]. The collagen production was affected by the duration of iontophoresis and pulse types of electric stimulation. The frequent-reversal bipolar electric stimulation was more effective than a continuous unipolar pulse electric stimulation at increasing skin collagen content.

Skin pretreatment with lasers was shown to promote the transdermal delivery of AA derivatives, such as 3-*O*-ethyl ascorbic acid and ascorbic acid 2-*O*-glucoside [108]. When the stratum corneum layer in the skin female Balb nude mouse was partly ablated by erbium:YAG laser treatment, the flux of 3-*O*-ethyl ascorbic acid and ascorbic acid 2-*O*-glucoside was 105 to 189-fold and 35 to 78-fold higher, respectively, than their flux across intact skin. When the skin was treated with CO_2_ laser, the flux of 3-*O*-ethyl ascorbic acid and ascorbic acid 2-*O*-glucoside was 181 to 277-fold and 82 to 117-fold higher, respectively, than their flux across intact skin.

Vibrating microneedles have been used to apply a 1.5% AA gel [109]. The vibrating microneedles enhanced the permeation of AA through the excised abdominal skin of the rat, in a manner dependent on the vibration intensity, application power, and AA gel application time [109]. Mesotherapy, a minimally invasive procedure involving a series of gentle, multiple micro-injections into the mesodermal layer under the skin, could be an additional option [110,111].

Photoacoustic waves, generated by laser pulses absorbed by piezophotonic (light-to-pressure) transducers, have been used to perturb the skin barrier and enhance skin delivery of ascorbyl 2-*O*-glucoside [112]. Exposure to photoacoustic waves for 5 min in combination with 2% ascorbyl 2-*O*-glucoside gel enhanced the drug delivery through the 760 μm thick pig skin samples by a factor of 15-fold with respect to passive delivery for 1 h contact of the formulation with the skin.

Thus, the use of medical devices or procedures has the advantage of enhancing the skin absorption of AA or its derivatives in a single- or multi-component preparation. However, medical devices or procedures would have limited access to ordinary consumers or patients if the devices are expensive or require technical skill in using them. In addition, caution is required because there is a risk that the use of electronic or mechanical devices causes side effects.

### 6.4. Antioxidant Effects of AA and Combination with Other Antioxidants

In the process of the interaction of internal and external factors, the skin undergoes various types of functional and structural degeneration associated with aging [18]. To delay or alleviate skin aging, the nutrition, energy, and redox balance of cells must be maintained in an optimal state [113]. For this purpose, the external supply of antioxidants such as AA is one of the attractive antiaging strategies [29]. AA (100 and 500 mM) treated on reconstituted human epidermis before or after UV irradiation at 120 mJ cm^−2^ attenuated cell death, apoptosis, ROS production, and tumor necrosis factor-α expression [114].

As an antioxidant, AA can reduce the breakdown of collagen directly or indirectly. MMPs are zinc-containing endopeptidases involved in the degradation of collagen and other ECM components [15,16]. The activities of MMPs increase in aged skin [23,24,66]. ROS stimulates activator protein-1 and nuclear factor-κB, which in turn stimulate the expression of MMPs in epidermal keratinocytes and dermal fibroblasts exposed to environmental factors, such as UV and particulate matter [19,115,116,117]. AA was shown to attenuate the expression of MMP 1 and 2 induced by UV-A [118] or that of MMP1 and MMP 9 induced by the combination of UV-A and particulate matter in human dermal fibroblasts [119]. The effect of AA inhibiting the expression of MMPs is considered to be associated with its antioxidant and anti-inflammatory effects [120].

The antioxidant effect of AA will be enhanced when it is combined with other antioxidants with different redox potentials, such as α-tocopherol [121,122]. The combination of AA with other vitamins, natural products, or peptides with different mechanisms of action synergistically protects cells from photooxidative stress [123], enhances collagen synthesis [72,73,74], and retards skin aging [71,75,76,77]. Since GSH and NADPH are used as electron donors for the enzymatic regeneration of AA, the biological effects of AA could be enhanced by combining AA with thiol compounds and nicotinamide. Research on the use of thiol compounds and nicotinamide as cosmeceuticals is actively underway [113,124].

The reason why various antioxidants with different redox potentials, mechanisms of action, and origins are used in combination is that it is expected that the biological effect will be better than when each antioxidant is used alone [125,126]. This is logical reasoning, but must be proven experimentally and clinically, because many antioxidants can act as pro-oxidants in some cases and increase oxidative stress in cells [127]. In addition, when antioxidants with chemical activity are formulated in a cosmetic preparation, they can affect the stability of the product [128]. Because prescribing multiple ingredients together has these advantages and disadvantages, caution is required in the selection of antioxidants to be combined with each other.

### 6.5. Combination of AA with Amino Acids to Synergistically Increase Collagen Production

Because ECM proteins, such as collagen and elastin, have unique amino acid compositions, their production in cells is influenced by the availability of specific amino acids [129,130,131,132]. Collagen proteins exist in the form of a triple helix, and the amino acid sequences repeat glycine-proline-X or glycine-X-hydroxyproline, where X is any amino acid [10]. Glutamine or glutamate increases proline production via pyrroline 5-carboxylate, and thereby collagen production in cells [133]. External proline supplementation increases collagen production, especially in glutamine-deficient media [134].

Because glycine residues occupy 1/3 of amino acid residues in collagen protein [10,13], the supply of glycine may be a limiting factor for collagen synthesis. A study by Paz-Lugo et al. [135] demonstrated that external glycine had a stronger effect on collagen production than proline, lysine, and other amino acids in articular chondrocytes. In our study [136], glycine enhanced collagen production most effectively among 20 different free amino acids in human dermal fibroblasts. In addition, glycinamide was the most effective among 20 different amidated amino acids. A surprising new finding was that glycinamide increased collagen production much more effectively than glycine. In contrast, other glycine derivatives, such as *N*-acetyl glycine, *N*-acetyl glycinamide, glycine methyl ester, glycine ethyl ester, and glycyl glycine, did not increase it. Thus, glycinamide is suggested to be an optimized form of glycine precursor to enhance collagen production in cells.

The combination of AA (1 mM) with glycinamide (1 mM) synergistically enhanced collagen production in human dermal fibroblasts to similar levels in cells treated with TGF-β1 (10 ng mL^−1^) [136]. AA derivatives, such as ascorbyl 2-phosphate (Magnesium salt), 3-*O*-ethyl ascorbic acid, ascorbyl tetraisopalmitate, and ascorbyl 2-*O*-glucoside, enhanced collagen production and showed a synergistic effect when treated in combination with glycinamide. Thus, it is possible to synergistically improve collagen synthesis by combining AA analogs (i.e., AA and ascorbyl 2-phosphate) and glycine analogs (i.e., glycine and glycinamide) [136].

The characteristic of combination therapy is that, instead of using one compound at a high concentration, several compounds with different mechanisms of action are combined at low concentrations to achieve the same or higher clinical effect [137,138]. Considering that cell collagen production can be enhanced in the transcriptional or translational stage according to changes in the cellular microenvironment, the combination of the several compounds acting at different stages is expected to have the advantage of synergistically increasing collagen production in cells [21]. It also has the advantage of avoiding the potential risk of side effects that each compound may cause at high concentrations [139]. However, the clinical utility of this combination therapy has not been established yet.

## 7. Discussion

Both intrinsic aging and extrinsic aging of the skin are mediated by oxidative stress and are accompanied by a decrease in the synthesis of ECM components and an increase in their decomposition. Therefore, the function of AA as an antioxidant and an enzyme cofactor is very important in maintaining skin health and preventing skin aging [28,29].

The results of several clinical trials suggest that AA and its precursor reduce wrinkles and increase the elasticity of the skin by preventing the loss of collagen in the process of photoaging and the natural aging of the skin [67,68,69,89]. External supplementation of AA can have a beneficial effect especially when the level of AA in the body is significantly lower than the normal state [67]. The skin collagen-enhancing effect of AA can be more apparent in patients with low collagen levels and people with photoaged or naturally aged skin [68]. On the other hand, when there is a sufficient amount of AA in the body, its supplementation may not be necessary and excessive administration could harm health [140]. Therefore, if the diagnosis of skin AA and collagen levels precedes, it is expected that the best clinical effect will be obtained by applying AA products customized to the patient.

The mechanism of action of AA promoting the production of functionally and structurally mature collagen is well established [53,54,55]. AA acts as a cofactor of 2-oxoglutarate-dependent dioxygenases, such as prolyl 4-hydroxylase, prolyl 3-hydroxylase, and lysyl 5-hydroxylase, which catalyze the hydroxylation of proline and lysine residues of procollagen [5,43]. On the other hand, although the action of the pharmacological dose of AA inducing the expression of collagen genes was discovered early in the 1980s [57,58], the detailed molecular mechanism remains to be further explored. Depending on the concentration, AA can function as either an antioxidant or a prooxidant and can directly activate transcription factors that control collagen gene expression by affecting cell signal transduction processes. Alternatively, AA can increase the expression of TGF-β1 and activate the related signal transduction process to finally increase the gene expression of collagen. A comprehensive study examining these possibilities is needed.

In the process of collagen production, AA plays a critical role in the gene transcription stage and the post-translational modification stage but does not have a direct effect on the translation stage. On the other hand, the supply of amino acids can have a significant impact at the translation stage by providing the building blocks of proteins. Thus, it is possible to synergistically increase collagen production by combining AA and amino acids that act at different stages of the collagen production process. Our recent in vitro study using human dermal fibroblasts fully supports this notion [136]. The strategy to enhance cellular collagen production using AA analogs (such as AA and ascorbyl 2-phosphate) in combination with amino acid analogs (such as glycine and glycinamide) would be useful for skin antiaging purposes. Further studies are needed to verify the clinical utility and efficacy of this combination strategy.

## 8. Conclusions

The chemical instability of AA and the difficulty of skin absorption have limited its medical and cosmetic applications. To solve these problems and attain the highest efficacy, various strategies are being developed. The dermal collagen-enhancing and skin antiaging effects of AA are more evident in patients with low AA and collagen levels. The combination of AA analogs with amino acid analogs may open a new possibility to effectively enhance skin collagen production for antiaging purposes. Further in vivo and clinical studies are needed to validate this new strategy.
